# Impacts of arbuscular mycorrhizal and *Trichoderma viride* on enhancing physicochemical properties and triggering defense mechanisms of tomato plants challenged with potato virus Y

**DOI:** 10.3389/fpls.2025.1650871

**Published:** 2025-08-22

**Authors:** Dalia Gamil Aseel, Omar M. Ibrahim, Toufic Elbeaino, Abdulaziz A. Al-Askar, Ahmed Abdelkhalek

**Affiliations:** ^1^ Plant Protection and Biomolecular Diagnosis Department, Arid Lands Cultivation Research Institute, City of Scientific Research and Technological Applications, Alexandria, Egypt; ^2^ Plant Production Department, Arid Lands Cultivation Research Institute (ALCRI), City of Scientific Research and Technological Applications, Alexandria, Egypt; ^3^ Department of Integrated Pest Management of Fruit Trees and Vegetable Crops, Istituto Agronomico Mediterraneo di Bari, Bari, Italy; ^4^ Department of Botany and Microbiology, College of Science, King Saud University, Riyadh, Saudi Arabia; ^5^ Plant Protection Department, The National Institute of Horticultural Research, Skierniewice, Poland

**Keywords:** arbuscular mycorrhizal, *Trichoderma viride*, PVY, defense genes, tomato

## Abstract

The utilization of arbuscular mycorrhizal fungi (AMF) and *Trichoderma* spp. correlates with improved plant nutrition and the stimulation of systemic plant defenses in response to pathogen challenges. Nonetheless, studies examining the effects of AMF colonization and the foliar application of the *Trichoderma viride* isolate Tvd44 on viral infection are limited. By analyzing the phenotypic, biochemical, and transcriptional expression of eleven defense genes, we investigated the effects of AMF colonization, foliar application of Tvd44, and their combined (dual) application on tomato plants challenged with potato virus Y. Interestingly, the dual application significantly suppressed viral symptoms and decreased viral accumulation levels, disease incidence, and disease severity by 88.1%, 40%, and 53.4%, respectively. Furthermore, both single and dual treatments significantly enhanced the activity of antioxidant enzymes, chlorophyll concentration, and macronutrient levels in the tomato tissues. In the realm of transcriptional analyses, the *CHS* gene served as a master key in understanding the physiological and pathway relationships among various genes (*F3’H*, *HQT*, *C3H*, *GST*, *JERF*, *CHI*, *WRKY-1*, *WRKY-19*, *FLS*, and *F3H*) involved in plant defense. These results suggest a sophisticated network of interactions that governs multiple facets of plant defense responses, encompassing the biosynthesis of flavonoids and other secondary metabolites, as well as the activation of transcription factors related to defense mechanisms. The obtained data indicate that AMF colonization and *T. viride* foliar spraying enhance tomato resistance to PVY by activating defense systems, thereby affecting viral replication. This finding highlights the significance of AMF and *T. viride* within the ecosystem and their crucial role in managing plant viruses.

## 1 Introduction

Viruses affecting plants lead to significant reductions in crop yield and quality, posing serious threats to global food security and the sustainability of agricultural practices (Rabie et al., 2024). Many viral infections cause widespread damage, ultimately leading to the death of the plant as the virus spreads throughout its system (Abdelkhalek and Hafez, 2019). Viral infections lead to necrosis, chlorosis, and abnormal growth patterns (Mandal et al., 2025), while they significantly diminish host plant performance by inhibiting photosynthetic carbon absorption and altering various processes, including the cell cycle, transport mechanisms, secondary metabolism, protein alterations, hormone regulation, and isoprenoid production (Abdelkhalek et al., 2019a). These alterations diminish plants’ protective mechanisms and facilitate virus proliferation. Potato virus Y (PVY, *Potyvirus yituberosi*, genus *Potyvirus*, family *Potyviridae*) is one of the most damaging viruses to potatoes and other solanaceous crops, such as tomatoes and peppers (Morais et al., 2025). The viral genome is single-stranded, positive-sense RNA of about 10 kb in length. It encodes a single large polypeptide, which is cleaved by three virus-encoded proteases into nine distinct products (Tribodet et al., 2005). Unlike fungi or bacteria, which may be treated with antifungal or antibacterial treatments, plants cannot be cured or controlled after contracting a virus. Consequently, the main goals of disease management are to prevent virus infection in plants or to increase plant resistance to viral infection. Although agrochemicals are commonly employed to manage plant virus infections by controlling their insect vectors, their elevated costs and potential environmental damage raise considerable concerns (El-Bilawy et al., 2022; Al-Askar et al., 2023).

Developing a sustainable approach is essential for managing viral infections and reducing the reliance on chemical fertilizers in agricultural areas. Consequently, the potential for improving plant immunity to viruses through the application of beneficial microbes, such as arbuscular mycorrhizal fungi (AMF), warrants careful consideration and has been suggested as a viable and sustainable approach to mitigate plant viruses (Aseel et al., 2019; Chaudhary et al., 2025). AMF assists plants in agricultural soils in overcoming biotic and abiotic challenges. Although morphologically restricted to the roots, AM fungi significantly influence the overall physiology of the plant due to the metabolic and physiological alterations they induce in the root system (Li et al., 2025). Their reliance on carbohydrates and lipids present in plants enables fungi symbionts to function as effective carbon sinks in roots. Therefore, plant carbon balance is preserved by regulating leaf primary metabolism and photosynthesis (Metwally and Abdelhameed, 2024). Setting up a functional AMF symbiosis also causes significant changes in the secondary metabolism of plants. These changes include alterations in the amount of phenolic compounds and the accumulation of phytohormones, such as jasmonic acid (JA), salicylic acid (SA), and abscisic acid (ABA) (Vasan et al., 2024). Despite evidence that AMF colonization mitigates the severity of diseases caused by several plant pathogens, it has received scant attention, and our comprehension of the influence of AMF on plant-virus interactions remains inadequate. The plant’s nutrition, the time of the encounter, and the lifestyle of the viral pathogen all influence the tripartite relationship between a plant, AMF, and a virus (Abid Mehmood et al., 2025).

Applying bio-inoculants containing *Trichoderma* as an antagonistic agent is among the most effective biological control methods in numerous countries (Ríos-Ruiz et al., 2025). The proportion of different *Trichoderma* species constitutes approximately 50-60% of the worldwide market for biological control agents (Yuan et al., 2025). *Trichoderma* induces local or systemic resistance in various plant pathogens, empowering plants to combat multiple diseases and enhancing overall plant productivity (Behiry et al., 2023). *Trichoderma* enhances the activation of defense responses in leaf tissue through the mediation of jasmonic acid (JA) (Aseel et al., 2023b). It produces enzymes and metabolites that can alter the ethylene levels in a plant’s root structure, enhancing nutrient uptake. Furthermore, it has the potential to bolster plant resistance, improve nutrient utilization efficiency, mitigate disease, stimulate plant growth, and remediate agrochemical contamination (Rodríguez-Martínez et al., 2025). The beneficial relationship between *Trichoderma* and plants can also induce the expression of genes related to plant defense mechanisms. Recently, it was reported that WRKYs regulate plant signaling by physically interacting with proteins involved in signaling, defense, transcription, and other cellular functions (Wang et al., 2025). Although the understanding of WRKY transcriptional activity during plant virus infection remains unclear, recent evidence suggests that WRKY transcription factors protect plants against virus infection (Nunna et al., 2025).

This investigation aimed to determine whether the combination of AMF and *Trichoderma viride* isolate Tvd44 enhances the resistance of tomato plants to biotic stress induced by PVY infection. Specifically, we aim to a) examine the impact of AMF on the colonization of tomato roots and/or the foliar application of Tvd44 to mitigate disease incidence and enhance tomato plant growth; b) investigate the transcriptomic expression of defense pathways, including *WRKY* transcription factors (*WRKY1* and *WRKY19*), jasmonic acid/ethylene (JA/ET) signaling like Jasmonate and ethylene-response factor 3 (*JERF3*) and Glutathione S-transferase 1(*GST1*), flavonoids such as Flavanone 3-hydroxylase (*F3H*), Chalcone synthase (*CHS*), Flavonol synthase 1 (*FLS*), Chalcone isomerase 2 (*CHI2*), and Flavonoid 3′ hydroxylase (*F3'H*), and chlorogenic acid like Hydroxycinnamoyl-CoA quinate transferase (*HQT*) and p-coumarate 3-hydroxylase (*C3H*) genes in tomato plants in response to PVY infection and c) analyze the expression of secondary metabolites and the physiological traits network of plants inoculated with AMF, Tvd44, and/or dual treatments co-inoculated with both fungi, against PVY.

## 2 Materials and methods

### 2.1 Tomato cultivar and virus inoculum source

The Agriculture Research Center in Egypt supplied a virus-free tomato (*Solanum lycopersicum*) cultivar, GS-12, showing susceptibility to PVY infection. The PVY strain DA55 employed in this research has been previously documented (Aseel et al., 2023b). The purified PVY served as the viral inoculum for all treatments challenged with PVY. The concentration of PVY inoculum was 20 µg/mL, prepared in a 10 mM phosphate buffer at pH 7.2, with the addition of 0.1% sodium sulfite.

### 2.2 Fungal inoculum

AMF was kindly obtained from the Plant Pathology Research Institute, Agricultural Research Centre, Egypt. The inoculum used in this study was made up of equal amounts of spores from *Rhizophagus irregularis* (Blaszk., Wubet, Renker, and Buscot), *Rhizoglomus clarum* (Nicolson and Schenck), and *Funneliformis mosseae* (Nicolson and Gerd.). We cultivated the AMF inoculum in sterile soil beneath Sudan grass (*Sorghum bicolor* L.) as a potential host. The AMF inoculum, exhibiting 83.1% colonization, consists of root fragments, mycelia, spores, and rhizospheric soil. The *T. viride* isolate Tvd44 (Ac# OQ991378) utilized in this study was previously isolated and characterized (Aseel et al., 2023b). The Tvd44 culture filtrate (spraying solution) was prepared by inoculating 1 mL containing 1 × 10^9 conidia into 100 mL of potato dextrose broth and culturing it at 28°C for 6 days on a rotary shaker at 150 rpm. Next, we filtered the culture by using Whatman filter paper No. 1. The filtrate was subjected to a 0.2 μm pore biological membrane filter before application to the plant leaves.

### 2.3 Antiviral assay and experimental design

Before planting, tomato seeds were surface-sterilized with a 70% ethyl alcohol solution and a 0.5% sodium hypochlorite (NaOCl) solution. At 20 days after seeds were planted in sterilized peat moss soil, tomato seedlings were transferred to 25-cm-diameter pots filled with a mixture of equal parts sterilized soil, sand, and clay, with five seedlings in each pot. Each treatment had five replicates. Fertilization was not applied, and all pots received regular irrigation. The plants were grown in a controlled greenhouse environment maintained at 26-28°C during the day and night, with a humidity level of 65%. Seven treatments were administered. The first treatment (control) consisted of tomato plants that were foliarly treated with sterilized medium-free Tvd44 and mechanically inoculated with viral inoculation buffer. The second treatment (AMF) consisted of tomato plants colonized by AMF, supplemented with a foliar application of sterilized medium-free Tvd44, and mechanically inoculated using a viral inoculation buffer. The third treatment (Tvd44) consisted of tomato plants that were foliarly treated with *T. viride* culture filtrate and mechanically inoculated with viral inoculation buffer. The fourth treatment (PVY) included tomato plants that were foliarly treated with sterilized medium-free Tvd44 and mechanically rubbed with PVY. The mechanical inoculation involved dusting the upper two leaves of each tomato seedling with carborundum and gently rubbing with 1 mL of PVY using the forefinger technique (Omar et al., 2022). The fifth treatment (AMF+PVY) consisted of tomato plants colonized with AMF, foliarly treated with sterilized medium-free Tvd44, and mechanically inoculated with PVY. The sixth treatment (Tvd44+PVY) included tomato plants that were foliarly treated with Tvd44 culture filtrate and mechanically inoculated with PVY. The seventh treatment (dual+PVY) involved tomato plants colonized by AMF, foliarly treated with *T. viride* culture filtrate, and mechanically inoculated with PVY. In all AMF-treated plants, AMF was applied during the transfer of tomato seedlings into new pots (at 20 days post-germination) by incorporating 10 g of the AMF inoculum into each seedling bed. The viral inoculation was conducted on all PVY-inoculated plants fifteen days after seedling transplantation, corresponding to 35 days post-germination. In every instance of Tvd44-treated plants, the culture filtrate was administered to the tomato leaves 24 h before the inoculation with PVY, corresponding to 34 days post-germination (at two fully expanded true leaves). A handheld pressure sprayer was employed to apply culture filtrate or sterilized medium-free Tvd44 to the entire plant until runoff occurred. All plants were maintained in insect-proof greenhouses for more than three weeks, and the manifestation of viral symptoms was monitored daily (Abdelkhalek et al., 2022a).

### 2.4 Sample collection and disease estimation

Tomato plants were collected 25 days post-PVY inoculation (dpi) and underwent various physiological and biomolecular assessments. The plants collected from each group were subjected to multiple washes with running water. For further analysis, three upper leaves from each plant were collected from every pot (15 leaves per pot) and categorized as a biological sample within the same treatment group, resulting in 5 biological samples. Every biological sample underwent three technical replicates. The disease incidence and severity were evaluated by observing visual viral symptoms, utilizing a rating scale from 0 to 5, and assessing the extent of leaf damage (Imran et al., 2012; Aseel et al., 2023b).

### 2.5 Mycorrhizal colonization estimation

Five tomato roots from each applied treatment were estimated for mycorrhizal colonization with PVY at 25 dpi. Small segments (1 cm) of each root were prepared and stained with trypan blue (Sigma-Aldrich, St. Louis, USA), as previously described (Phillips and Hayman, 1970). Mycorrhizal colonization was assessed in root segments of each treatment using a light microscope, adhering to the previously described approach (Trouvelot, 1986).

### 2.6 Growth parameter estimation

At 25 dpi, all plants from each treatment were meticulously uprooted, washed under running water, and assessed for the number of leaves, root and shoot lengths (in cm), and shoot and root dry weights (in g). The dry weights were measured following a 72-hour drying period of the plant samples in an oven set to 40°C.

### 2.7 Estimation of macronutrient content

The nitrogen (N) content in the tomato leaves was determined through the Kjeldahl method (Horneck and Miller, 2019). As previously described, total sulfur (S) was determined by precipitating the sulfate with barium chloride and measuring turbidity using a spectrophotometer at 420 nm (Olsen and Sommers, 1982).

### 2.8 Biochemical estimation

The evaluation of photosynthetic chlorophyll a and b in tomato leaves was conducted as described previously (Harborne, 1998). The techniques for extracting and assessing the enzymes peroxidase (POX), polyphenol oxidase (PPO), and catalase (CAT) were established by Maxwell and Bateman (1967); Galeazzi et al. (1981), and Aeby (1984), respectively. The extraction and methodology for quantifying total protein content were conducted following Bradford (1976).

### 2.9 Transcriptomic profiles of the defense pathway genes

Total RNA was extracted from 100 mg of fresh tomato leaves collected at 25 days post-inoculation (dpi) using the RNeasy Plant Mini Kit according to the manufacturer’s instructions. Following the RNA concentration and purity assessment, a reverse transcription procedure was conducted to convert one µg of DNase-treated RNA into cDNA utilizing M-MuLV reverse transcriptase, as previously outlined (Aseel et al., 2023b). The cDNA was stored at -20°C until it was used. The evaluation of AMF colonization and *T. viride* treatments on the expression of defense-related genes and the accumulation level of the *PVY-CP* gene in tomato plants was conducted using real-time quantitative PCR (RT-qPCR). The expression ratio of the *PVY-CP* gene to the housekeeping gene in tomato control plants was utilized to determine the level of viral accumulation. The sequences of specific primers for tomato defense pathway genes *WRKY1*, *WRKY19*, *JERF3*, *GST1*, *CHS*, *C3H*, *CHI2*, *FLS*, *F3H*, *F3'H*, and HQT, as well as PVY-CP, are listed in **Supplementary Table S1**. The *β*-actin gene was selected as a housekeeping gene due to its stable expression in tomatoes, as reported in prior studies (Aseel et al., 2023a; Rabie et al., 2024). The RT-qPCR experiment utilized a CFX Connect TM Real-Time System (BIO-RAD, USA). Each reaction contained 10 µL of 2xSYBR Green RT Mix (Bioline, Germany), 7.4 µL of RNase-free water, 0.8 µL of each primer (10 pmol/µL), and 1 µL of cDNA. The RT-qPCR protocol consisted of an initial cycle at 95°C for 5 min, followed by 45 cycles of 95°C for 5 seconds, 60°C for 10 seconds, and 72°C for 15 seconds. The relative gene expression was determined using the comparative C_T_ approach (2^−ΔΔCT^) as described previously (Schmittgen and Livak, 2008).

### 2.10 Statistical analysis

Volcano plots were created using the *ggplot2* package, and a t-test was employed to determine the statistical significance of gene expression compared to the control treatment. The significance was expressed as -log_10,_ while gene expression was expressed as log_2_ fold-change. Heatmap and cluster analysis figures of gene expression were generated using *pheatmap* package. The data were first transformed using log_2_; then, Euclidean distance was applied, followed by the Ward method of hard clustering. Thirty internal validation indexes were employed to determine the optimal number of clusters in the data (Charrad et al., 2014). Principal component analysis was conducted using function *prcomp* in *stats* package. The figures of contribution and biplot were built using the *factoextra* package. Gene expression and physiological trait networks were generated using the *qgraph* package, based on the Spearman rank correlation coefficient, to connect gene expression results with physiological data. All statistical analyses were performed using R software version 4.3.2 (2023).

## 3 Results

### 3.1 Viral symptoms development and disease assessment

**Figure 1:**
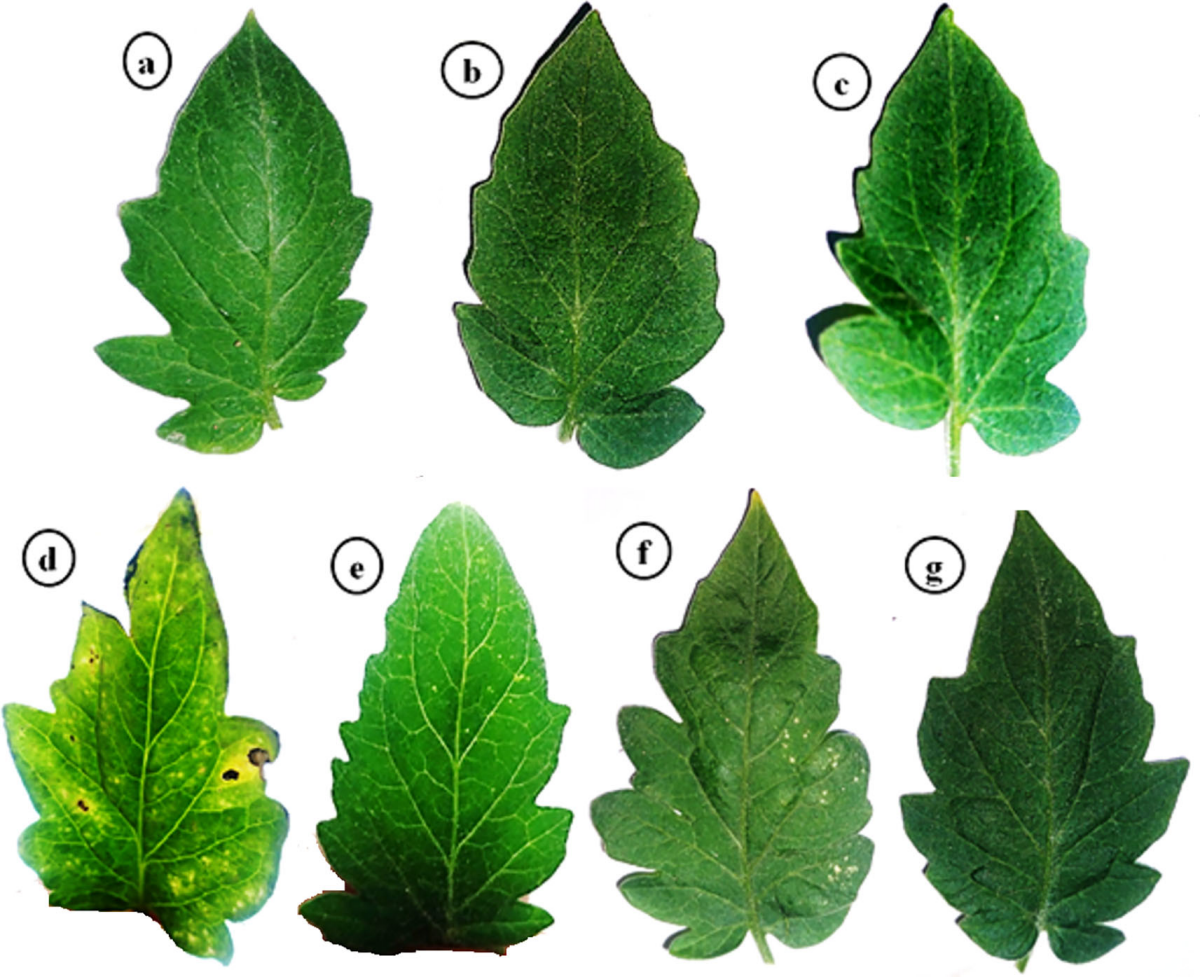
Impacts of arbuscular mycorrhizal and *Trichoderma viri*de on PVY symptoms at 25 dpi. **(a)** control plants, **(b)** AMF plants colonized with AM fungi, **(c)** sprayed with Tvd44, **(d)** PVY-infected tomato plants, **(e)** colonized with AMF and infected with PVY, **(f)** sprayed with Tvd44 and infected with PVY, **(g)** colonized with AMF and infected with PVY and sprayed with Tvd44.

**Figure 2:**
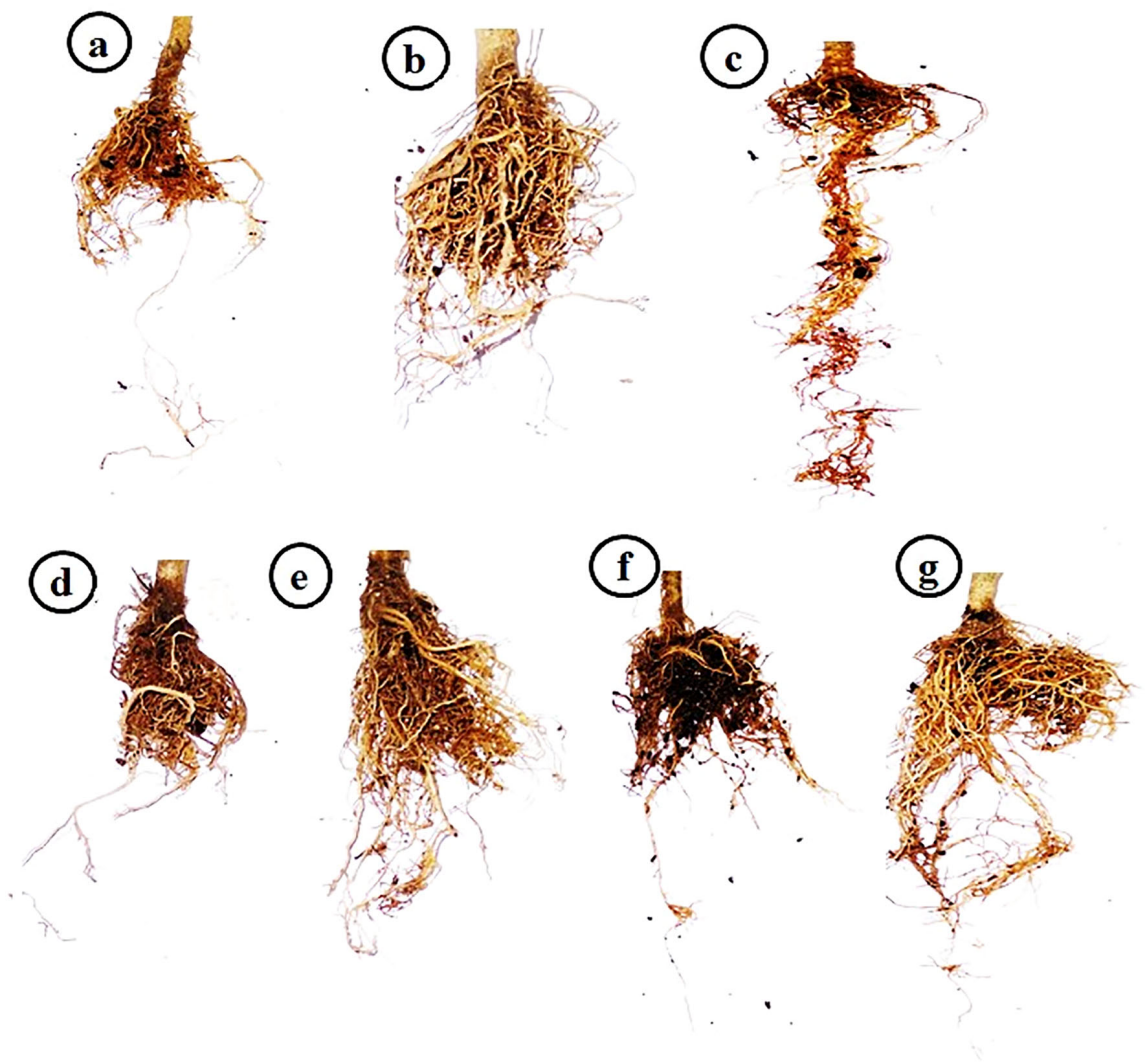
Impacts of arbuscular mycorrhizal and *Trichoderma viride* on tomato roots at 25 dpi. **(a)** control plants, **(b)** AMF plants colonized with AM fungi, **(c)** sprayed with Tvd44, **(d)** PVY-infected tomato plants, **(e)** colonized with AMF and infected with PVY, **(f)** sprayed with Tvd44 and infected with PVY, **(g)** colonized with AMF and infected with PVY and sprayed with Tvd44.

Under controlled greenhouse conditions, the tomato plants treated with PVY exhibited characteristic viral symptoms at 19 dpi. The pronounced leaf mosaic, distinct veins, yellowing, and necrosis were distinctly observable at 22 dpi (**Figure 1**). Interestingly, the AMF+PVY, Tvd44+PVY, and dual+PVY treatments resulted in a delay of approximately 5 days in the appearance of symptoms, with mild symptoms observed at 25 dpi (**Figure 1**). No symptoms were observed in the control, AMF, and Tvd44 treatments. The findings demonstrated that the PVY treatment resulted in a 100% incidence of disease and a severity level reaching 96.8% (**Table 1**). The findings correspond to the accumulation level of the *PVY-CP* gene, indicating an approximately 27.8-fold increase in PVY-treated plants relative to control plants. Compared to PVV treatment, the AMF+PVY, Tvd44+PVY, and dual+PVY treatments exhibited significantly reduced PVY accumulation expressions, with relative expression levels of 4.82-, 5.84-, and 3.29-fold, respectively. The dual+PVY treatment resulted in a decrease in disease incidence and severity to 60% and 46.62%, respectively. The AMF+PVY and Tvd44+PVY exhibited disease severity levels of 51.96% and 54.64%, respectively, as shown in **Table 1**. Nonetheless, the data presented in **Figure 2** indicated that the roots of infected plants with PVY were shorter than those treated with AMF+PVY, Tvd44+PVY, and/or the dual+PVY treatments, which exhibited a significantly greater length.

**Table 1:** Evaluation of disease incidence and disease severity of tomato plants upon PVY challenge and treatment with AMF and Tvd44 at 25 dpi.

Treatments	Disease incidence		Disease severity	
	Value %*	Decrease %	Value %	Decrease %
PVY	100 a	0	96.8 ± 3.34 a	0
AMF+PVY	60 c	40	51.96 ± 5.61 c	48.04
Tvd44+PVY	64 b	36	54.64 ± 2.99 b	45.36
dual+PVY	60 c	40	46.62 ± 4.71 d	53.38

*The means in each column that share the same letter do not show significant differences at a probability level of 0.05. The standard deviation (SD) expressed as ±.

### 3.2 Mycorrhizal colonization estimation

**Figure 3:**
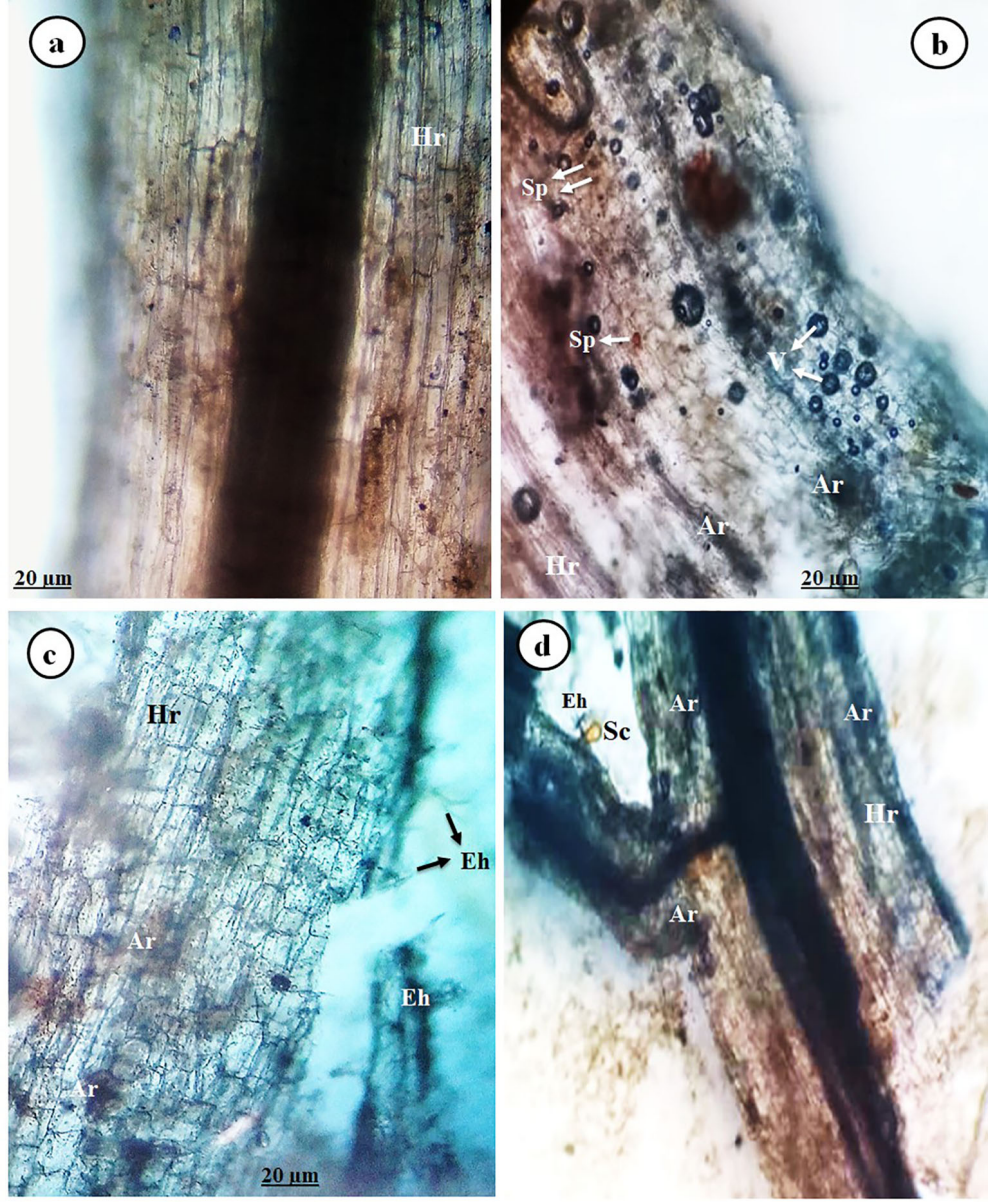
Image showing light micrographs of tomato roots colonized with AMF displaying typical mycorrhizal structures (at 25 dpi), control root **(a)**, and AMF-colonized tomato roots **(b–d)**, where Hr, host root; Ar, arbuscule; Eh, exteraradical hyphae; Sc, sporocarp; Sp, spore; and V, vesicle.

Concerning mycorrhizal colonization in tomato roots infected with PVY and/or treated with Tvd44 at 25 dpi, the data revealed that no mycorrhizal colonization was detected in tomato plants that did not receive the AMF inoculum. On the contrary, the other treatments with AMF inoculum showed varying degrees of mycorrhizal colonization. Different typical mycorrhizal structures were observed in the tomato roots through microscopic examination (**Figure 3**). Successful colonization was confirmed by light microscopy investigation, which revealed the presence of both arbuscules and intraradical mycelium in the root cortex of the colonized plants (**Figures 3b–d**). Finally, as indicated by our results, PVY-uninfected tomato plants treated only with AMF inoculum exhibited the highest degree of colonization frequency (83.1%).

### 3.3 Growth parameters evaluation

**Figure 4:**
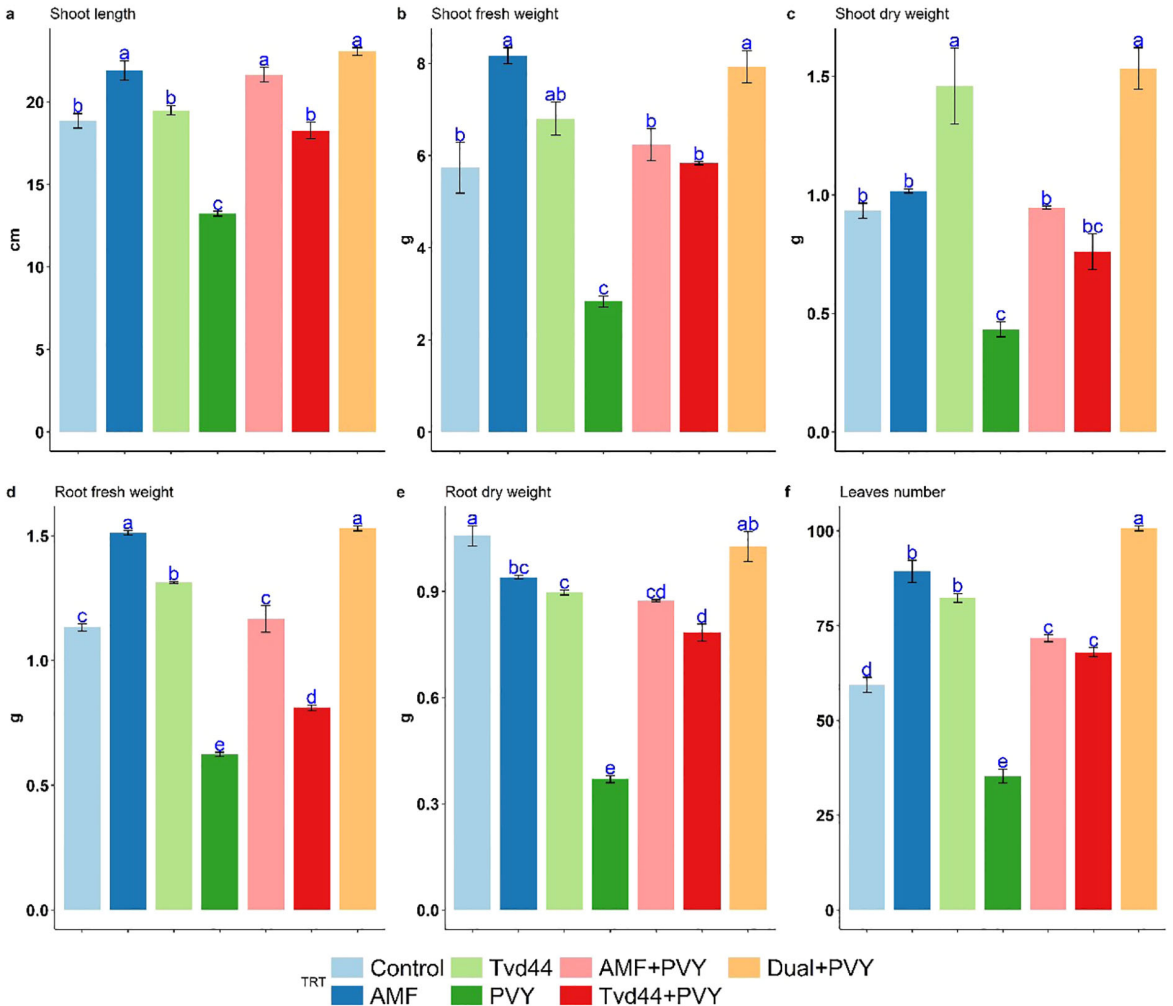
Impact of 7 treatments at 25 dpi on shoot length (cm) **(a)**, shoot fresh weight (g) **(b)**, root fresh weight (g) **(c)**, shoot dry weight (g) **(d)**, root dry weight (g) **(e)**, and leaf number **(f)** traits using Tukey's HSD test at p ≤ 0.05. Statistical significance was indicated alphabetically above the histogram in ascending order, whereas a>b>c>d. The means in each column that share the same letter do not show significant differences.

**Figure 4** displays the mean growth parameters of 25 dpi tomato plants in response to the various treatments. More precisely, treating tomato plants with AMF and foliar Tvd44 improved their shoot height, root length, fresh weight (both root and shoot), dry weight (both root and shoot), and leaf number compared to control tomato plants. Meanwhile, AMF root colonization promoted all assessed growth parameters. In contrast, PVY treatment significantly decreases all assessed growth parameters compared to control tomato plants. The colonization with AMF, foliar Tvd44, and dual treatments reduces the adverse impacts of PVY infection on the estimated parameters compared with untreated infected plants. Compared to PVY treatment, the dual+PVY treatment was more efficient than the AMF colonization and foliar Tvd44 alone. The dual+PVY treatment resulted in a significant increase of 180% and 254% in shoot and root fresh weight, respectively; additionally, leaf numbers increased by 185% (**Figure 4**).

### 3.4 Effect of treatments on macronutrient content and biochemical traits

**Figure 5:**
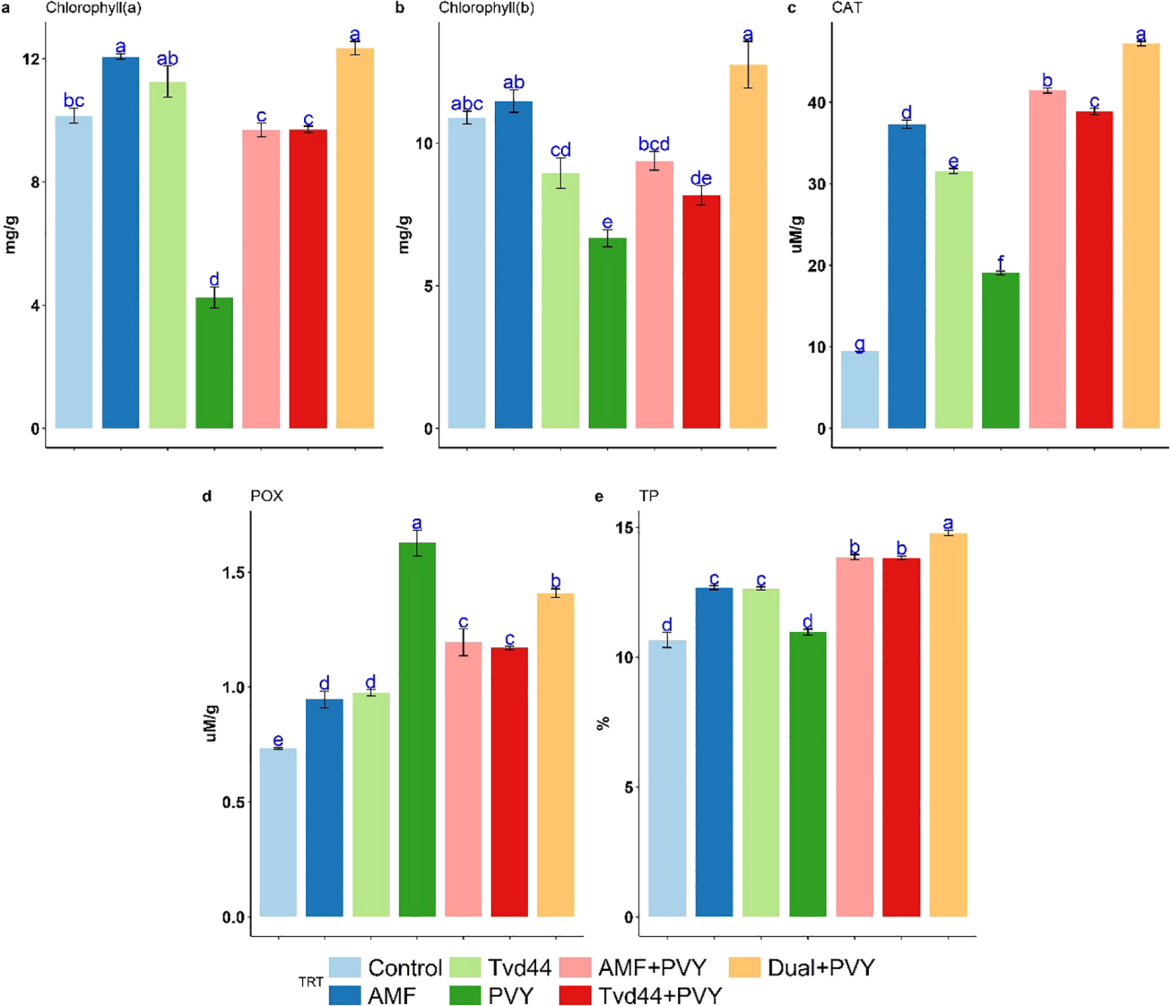
Impact of 7 treatments at 25 dpi on chlorophyll **(a)**, chlorophyll **(b)**, catalase (CAT) **(c)**, peroxidase (POX) **(d)**, and total protein (TP) **(e)** traits using Tukey's HSD test at p ≤ 0.05. Statistical significance was indicated alphabetically above the histogram in ascending order, whereas a>b>c>d. The means in each column that share the same letter do not show significant differences.

The total nitrogen (TN) content in tomato leaves varied among the different treatments (**Table 2**). All treatments resulted in increased TN levels compared to the control treatment. The most significant increases in TN content were noted in the dual+PVY treatment at 3.84%, followed by AMF at 3.45% and Tvd44 at 3.39%. AMF colonization reached its peak in total carbon content at 34.95%, closely followed by Tvd44+PVY at 33.95%. Moreover, the majority of the treatments implemented resulted in elevated levels of total hydrogen (TH) and total sulfur (TS). The observed C/N ratios decreased across all treatments compared to the control (**Table 2**). **Figure 5** presents the findings from the Tukey multiple comparison test concerning the effects of AMF and Tvd44 on biochemical traits. The dual+PVY treatment resulted in a significant increase in chlorophyll (a), (b), CAT, and TP compared to the PVY treatment, with enhancements of 191%, 91%, 147%, and 34%, respectively.

**Table 2:** Effect of AMF and *T. viride* isolate Tvd44 on the macronutrient contents in the tomato leaves.

Category	Treatments	Total nitrogen (%)*	Total carbon (%)	Total hydrogen (%)	Total sulfur (%)	C/N ratio (%)	C/H ratio (%)
Category 1	Control	2.35 d	33.8 b	5.30 a	1.29 a	14.4 a	6.37 c
	PVY	2.64 c	31.9 d	4.87 b	0.65 c	12.1 b	6.56 b
	AMF	3.45 b	34.9 a	5.28 a	0.61 d	11.7 c	6.62 a
	Tvd44	3.39 a	33.4 c	5.24 a	0.89 b	9.9 d	6.38 c
Category 2	PVY	2.64 d	31.9 d	4.87 d	0.65 d	12.1 a	6.56 a
	AMF+PVY	3.13 c	33.3 b	5.11 b	1.01 b	10.6 b	6.52 c
	Tvd44+PVY	3.35 b	33.9 a	5.21 a	0.94 c	10.1 c	6.52 c
	dual+PVY	3.84 a	32.1 c	4.91 c	1.08 a	8.34 d	6.53 b

*Each value in the table represents the mean of five replicates. The means in each column that share the same letter do not show significant differences at a probability level of 0.05.

### 3.5 Transcriptional levels of defense-related genes

**Figure 6:**
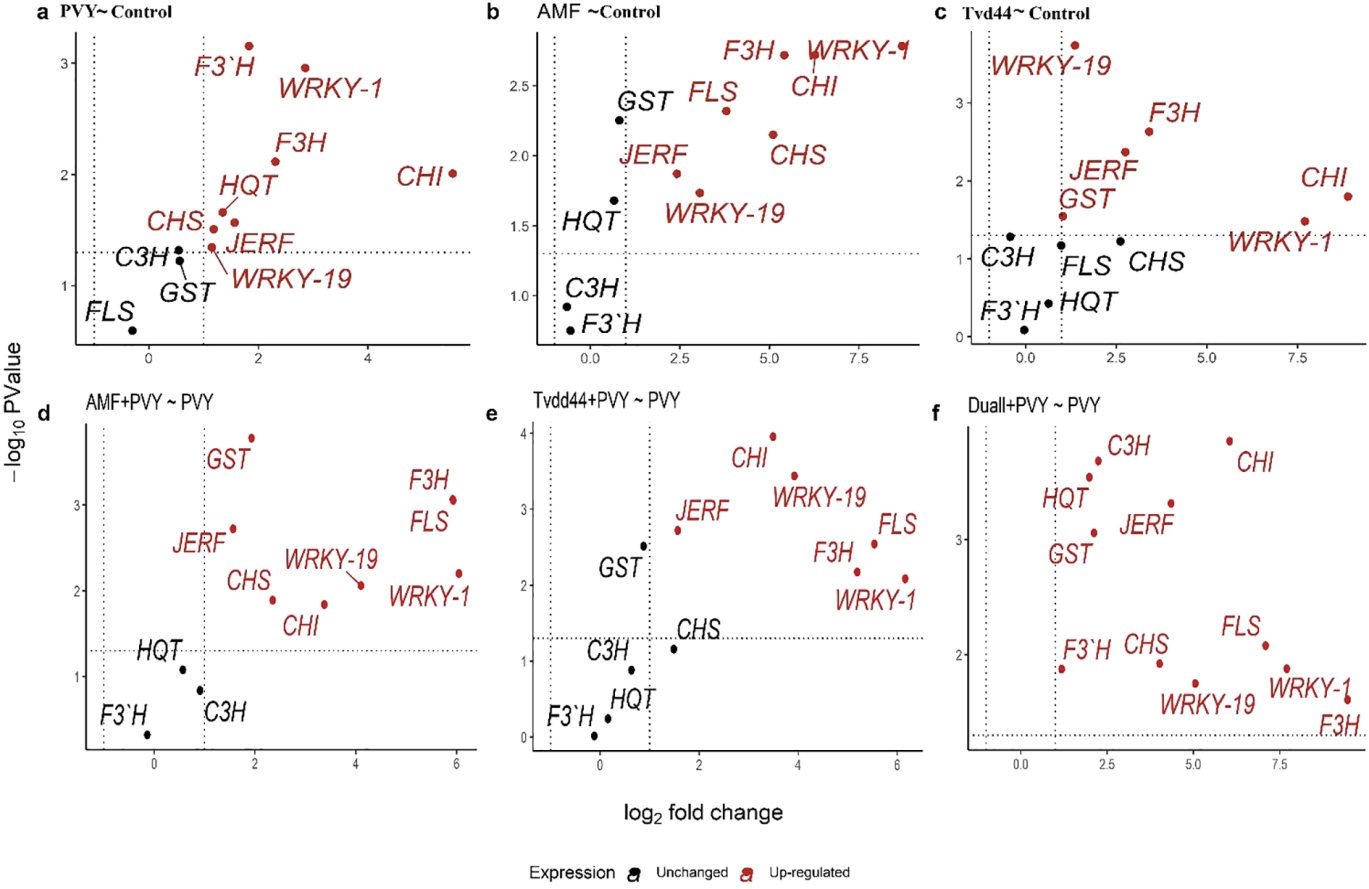
Volcano plots of change in gene expression for eleven genes in tomato leaves infected with PVY in response to AMF colonization and *T. viride* at 25 dpi. Where y-axis represents *P*-values (-log10), the x-axis represents fold change (log_2_), the black color represents unchanged genes, and the red color represents significantly upregulated genes. Because *P*-values on the y-axis in volcano plots were transformed and became negative, the higher the number on the y-axis, the smaller the *P*-value and the greater the significance. Threshold indicator dashed lines were drawn on volcano plots, where genes above the horizontal dashed line are significantly expressed at a *p*-value of 0.05. Genes beyond the right vertical dashed line are upregulated, genes behind the left vertical dashed line are downregulated, and genes between the two vertical dashed lines are unchanged. Relative gene expression changes among PVY and control **(a)**, AMF and control **(b)**, Tvd44 and control **(c)**, AMF+PVY and PVY **(d)**, Tvd44+PVY and PVY **(e)**, and Dual+PVY and PVY **(f)** treatments.

The present study assessed the relative expression levels of eleven genes involved in regulating the five key components of the defense pathways, using RT-qPCR at 25 dpi (**Figure 6**). The analysis revealed that the infection of tomato plants with PVY resulted in a 7.2-fold increase in the expression level of *WRKY1* compared to the control group. Furthermore, the treatments AMF+PVY and Tvd44+PVY applications exhibited the highest expression levels of *WRKY1*, 66.3- and 71.9-fold increase, respectively (**Figure 6**). The expression levels of *WRKY19* exhibited varying degrees of increase in response to PVY, AMF, and Tvd44 treatments compared to the control group. *JERF3* exhibited stimulation following infection with PVY, AMF, and Tvd44 treatments compared to control plants. Concurrently, the expression levels of AMF+PVY, Tvd44+PVY, and the dual+PVY treatment increased by 2.9-, 2.9-, and 20.2-fold, respectively (**Figure 6**). Concerning *GST1*, the infected plants underwent treatment with AMV and Tvd44, resulting in a significant increase in the transcript level of *GST1*. Notably, the dual+PVY treatment elicited a more pronounced response than the treatments involving infection alone. The findings from **Figure 6** indicate that all the treatments applied led to an increase in the expression levels of three genes: *F3H* and *CHI2* at 25 dpi, with notable enhancement in the AMF+PVY, Tvd44+PVY, and dual+PVY treatment groups. Conversely, the PVY treatment showed no impact on the expression of *FLS* gene transcripts. In contrast, treatments involving AMF+PVY, Tvd44+PVY, and dual+PVY induction resulted in transcript gene expression levels of 59.9-, 45.7-, and 134.9-fold, respectively. The data presented in **Figure 6** indicate no effect on the *F3'H* expression for the tomato plant treatments utilizing AMF or Tvd44 alone. However, an impact was noted for all applied treatments on the F3H gene expression, particularly in tomato plants infected with PVY, which exhibited a 3.54-fold increase. All applied treatments induce the expression of *HQT* gene transcripts in the chlorogenic pathway. In *C3H*, a downregulating effect was noted when treating AMV or Tvd44 individually, in contrast to all treatments infected with PVY, which increased gene transcript expression.

### 3.6 Statistical significance of gene expression

Results from the volcano plots in **Figure 6a** showed that infected plants with the virus led to significant upregulation of all the studied genes except for *GST1*, *C3H*, and *FLS*. When plants were treated with AMF, as shown in **Figure 6b**, seven out of eleven genes were significantly upregulated (*JERF3*, *WRKY1, WRKY19, CHS, CHI2, F3H*, and *FLS*), while four genes (*GST1, HQT, C3H*, and *F3’H*) were unchanged. In **Figure 6c**, when treatment Tvd44 was applied, six genes were significantly upregulated (*WRKY1, WRKY19, F3H, CHI2, JERF*, and *GST1*), while five genes were unchanged (*C3H, FLS, CHS, HQT*, and *F3’H*). **Figure 6d** revealed that in plants treated with AMF+PVY, eight out of eleven genes were significantly upregulated (*JERF3*, *WRKY1, WRKY19, CHS, CHI2, F3H, GST1*, and *FLS*), while three genes (*HQT, C3H*, and *F3’H*) were unchanged. When the treatment Tvd44 was applied before virus infection (Tvd44+PVY), as shown in **Figure 6e**, six out of eleven genes were significantly upregulated (*JERF3*, *WRKY1, WRKY19, CHI2, F3H*, and *FLS*), while five genes (*HQT, C3H, CHS, GST1*, and *F3’H*) were unchanged. The application of dual+PVY treatments resulted in a significant upregulation of all eleven studied genes (**Figure 6f**).

### 3.7 Heatmap and cluster analysis of the differentially expressed genes

**Figure 7:**
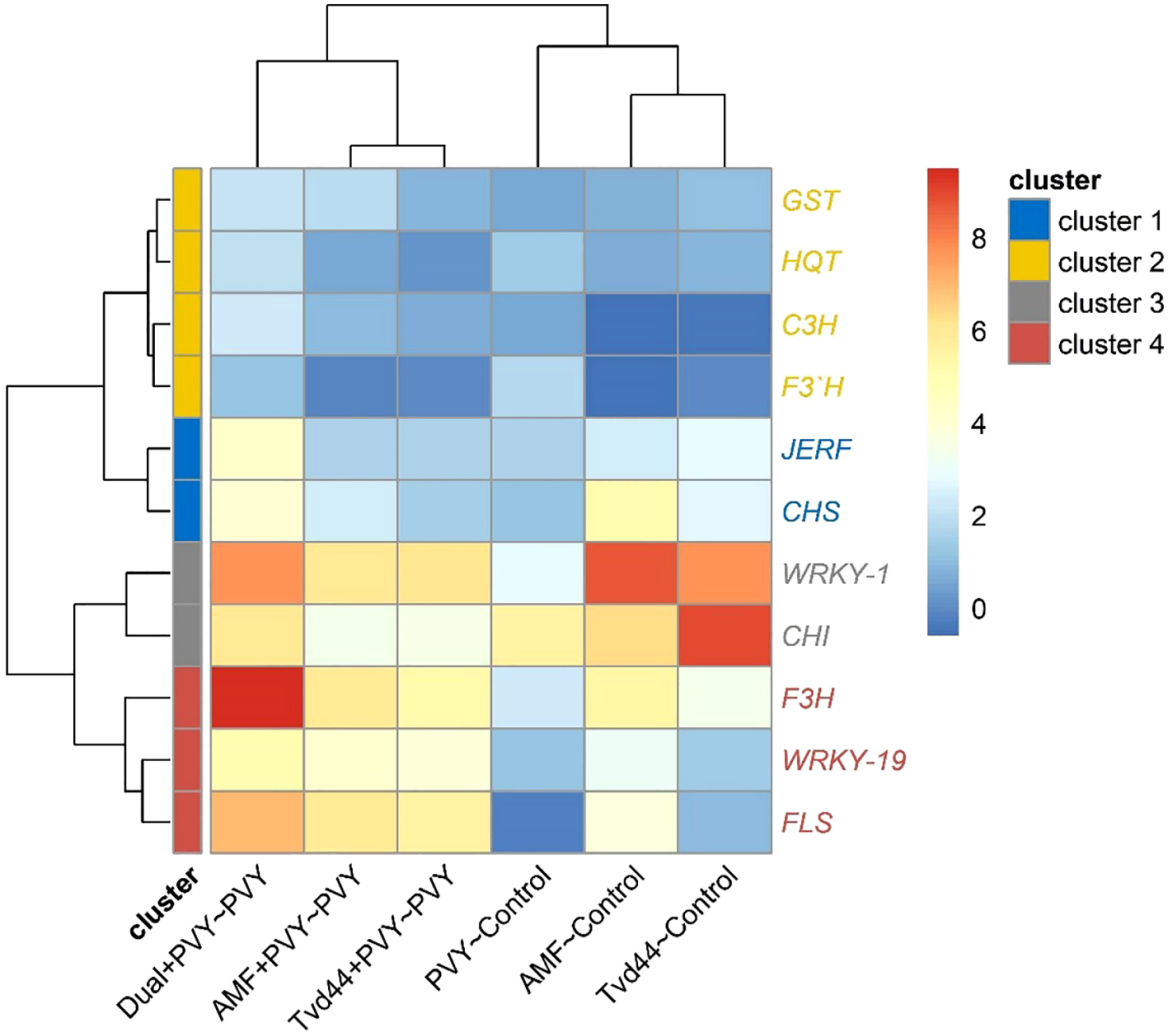
Heatmap and hierarchical clustering of change in gene expression for eleven genes in tomato leaves infected with PVY in response to AMF colonization and *T. viride* at 25 dpi. Cell color intensities were based on gene expression, represented as log_2_, where blue indicates downregulated genes and red indicates upregulated genes.

The heatmap in **Figure 7** was constructed based on two treatment groups. The first group consists of treatments with PVY, AMF, and Tvd44 against the control, while the second group comprises treatments with AMF+PVY, Tvd44+PVY, and dual+PVY against PVY. The heatmap demonstrates that the eleven studied genes were grouped into four clusters based on their expression. Four genes in the second cluster (*C3H*, *GST1, F3’H*, and HQT) exhibited similar expression patterns across all treatments, meaning they responded similarly to each treatment. In contrast, two genes (*WRKY1 and CHI2*) in the third cluster and three genes (*WRKY19*, *FLS*, and *F3H* in the fourth cluster exhibited different expression patterns at each treatment. For example, in the dual+PVY~PVY treatment, the genes of the third and fourth clusters exhibited different expression levels (represented by different colors), with *WRKY1* and *F3H* being highly expressed, followed by *CHI2*, *FLS*, and *WRKY19*. For the same treatment, the expression of the first cluster genes (*JERF* and *CHS*) was similar in their expression (same color). These results highlight the significant role of the third and fourth clusters of genes in response to PVY, compared to the first and second clusters.

### 3.8 Principal component analysis

**Figure 8:**
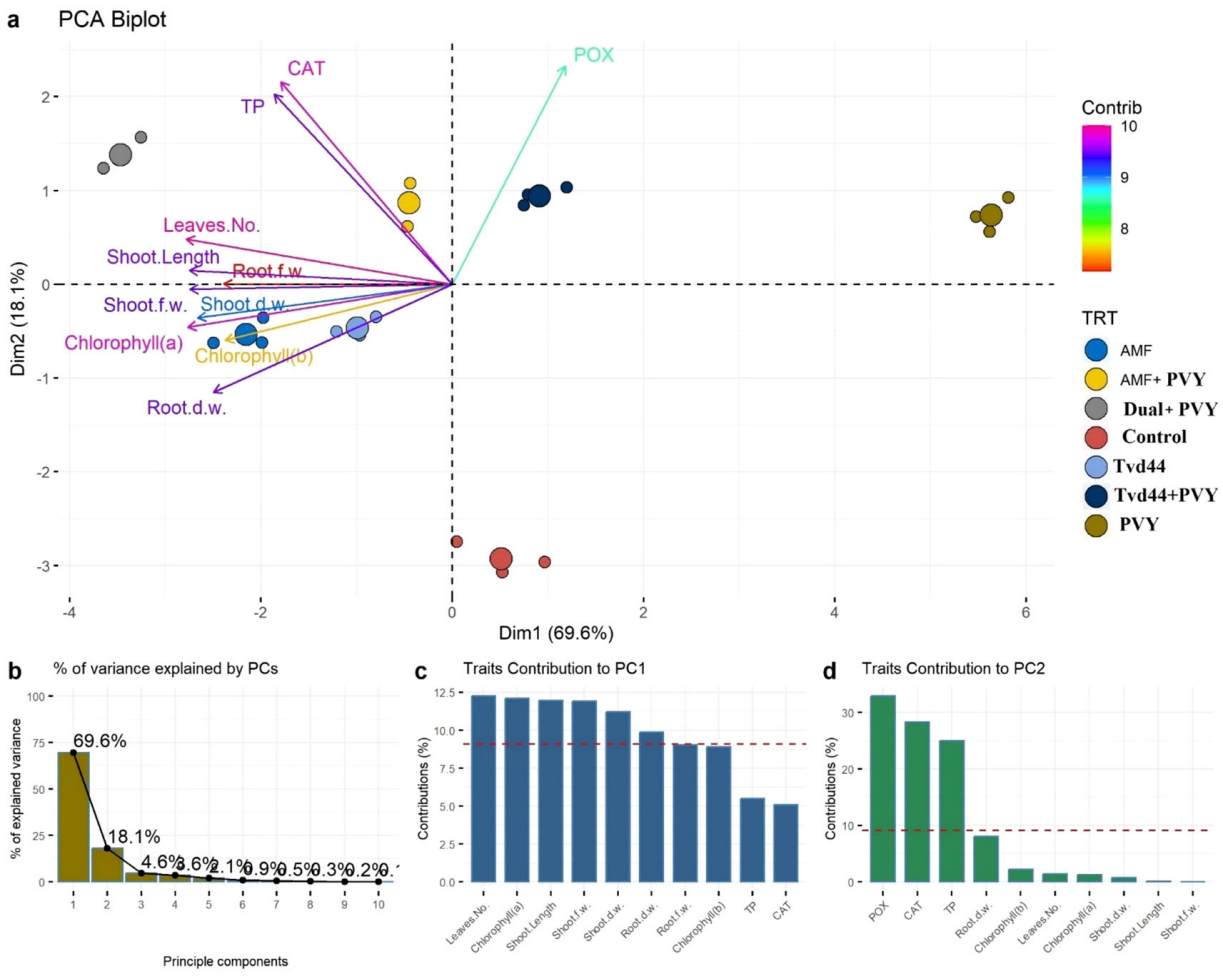
**(a)** Principal component analysis (PCA) biplot illustrates the contribution of each dimension to the total variance of 7 treatments and 11 physiological traits. Longer arrows specify higher contribution, while shorter arrows show lower contribution. **(b)** The bar plot indicates the percentage of contribution of PCs to the total variance. **(c, d)** bar plots of the contribution of the 11 studied traits to PCs where traits above the dashed red line specify significant contribution.

**Figures 8a, b** show that the first two principal components (PCs) account for 87.7% of the total variance, with 69.6% and 18.1%, respectively. The first principal component was represented by leaf number, chlorophyll (a), shoot length, shoot weight (both fresh and dry), and root dry weight, with higher loadings (longer arrows) located in the left area of the biplot. At the same time, the second principal component was represented by CAT, POX, and TP, which had higher loadings and were located in the upper center of the biplot (**Figures 8c, d**). These results illustrated that all traits were suitable for distinguishing between control and PVY treatments, as well as the rest.

### 3.9 Gene expression and physiological traits network

**Figure 9:**
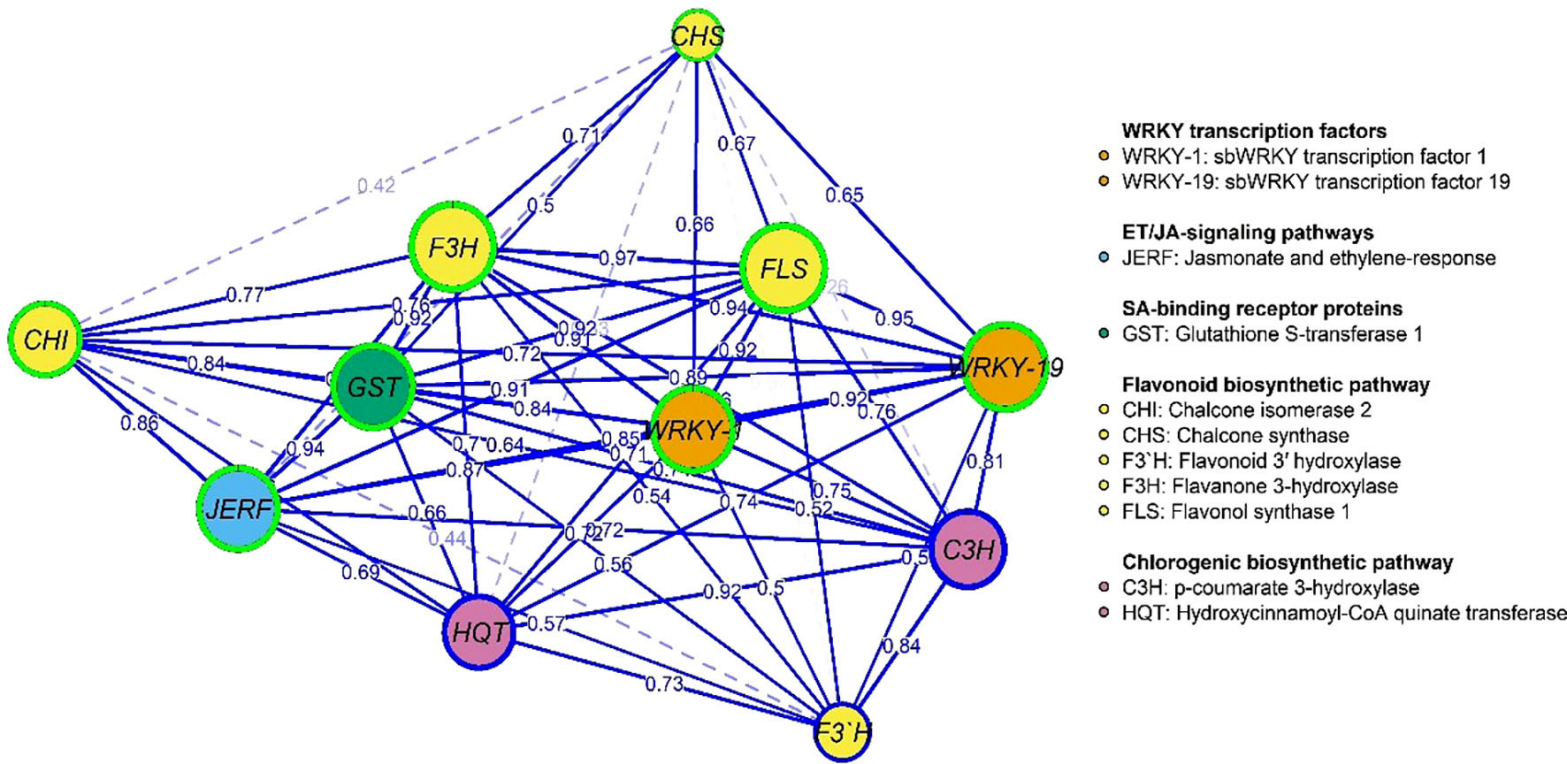
Gene co-expression network: nodes (circles) represent genes, while edges (lines) represent associations among genes based on the Spearman rank correlation coefficient. Faded lines represent non-significant correlations.

**Figure 10:**
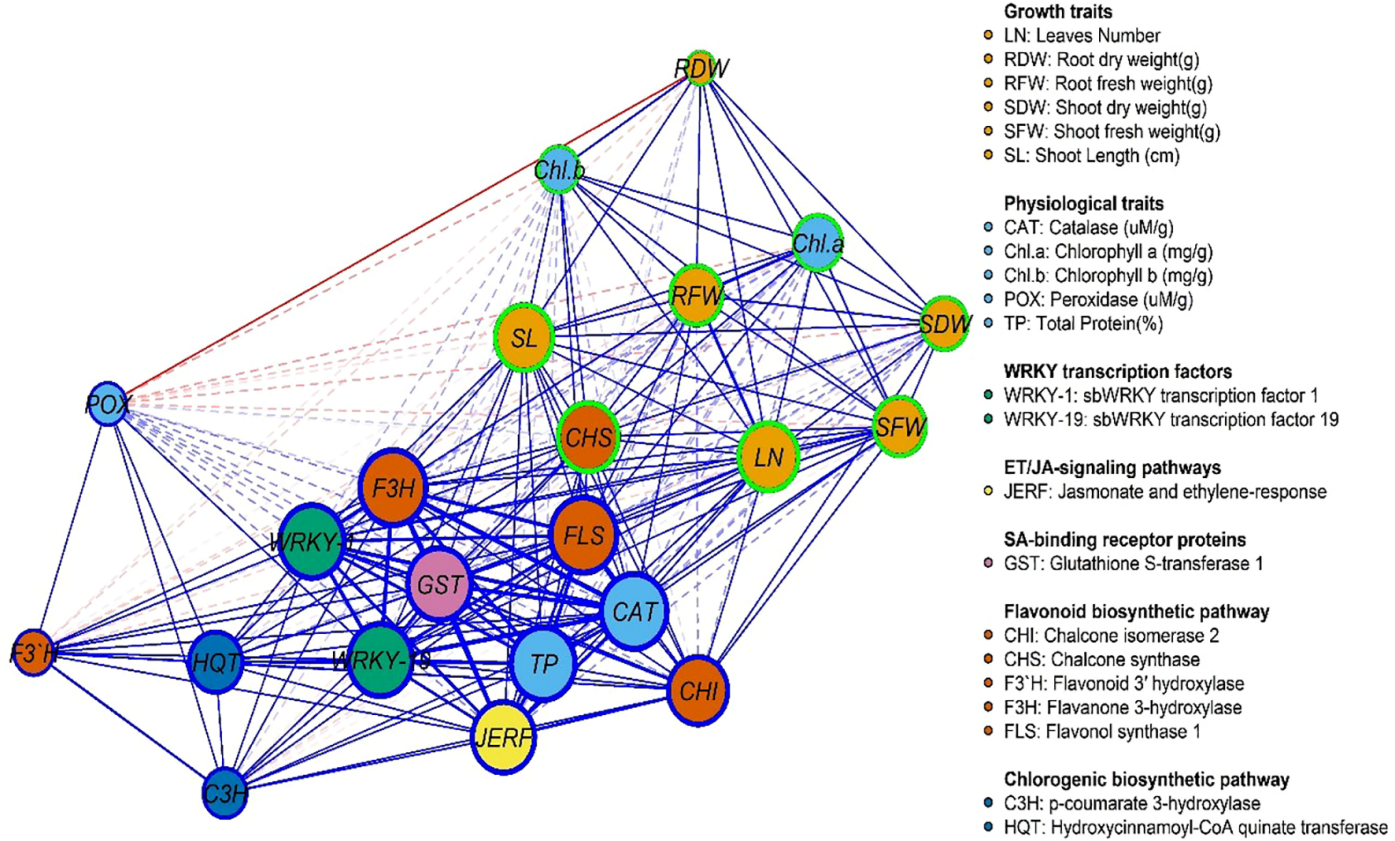
Gene and physiological traits network, where nodes (circles) represent genes and physiological traits, and edges (lines) represent associations among genes and physiological traits based on the Spearman rank correlation coefficient. Faded lines represent non-significant correlations.

**Figures 9**, **10** represent the network of eleven gene expressions and eleven physiological traits in response to the seven treatments. The network revealed that all eleven studied genes were significantly correlated, except for gene *CH3* with *JERF3*, *CHI2*, *HQT*, *C3H*, *F3’H*, and *CHI2* with *F3’H*. The least correlated physiological traits to both genes and other traits were root dry weight, POX, and chlorophyll (b). The most correlated were CAT, TP, and the number of leaves. On the other hand, the gene least correlated with both other genes and physiological traits was *F3’H*, while the most correlated ones were *F3H, FLS, WRKY1, WRKY19, GST1, JERF3*, and *CHS.* The most highly correlated gene with the physiological traits was *CHS*, which correlated with nine traits, followed by *FLS*, which correlated with eight traits, and *CHI2*, *WRKY19*, and *F3H*, which correlated with six traits each. *GST1* and *WRKY1* correlated with five traits, followed by *JERF3*, which correlated with four traits. Finally, *HQT, F3’H*, and *C3H* were the least correlated with the physiological traits, as they were correlated with only three traits. The network identified two local communities; the first community (nodes with blue borders) comprised 10 genes, excluding *CHS*, and three physiological traits (CAT, POX, and TP). The second community (nodes with green border) contained eight physiological traits, except for CAT, POX, TP, and one gene (*CHS*).

## 4 Discussion

The effectiveness of defense priming in a biological system has been assessed based on several key criteria: enhanced defense responses, retention, better outcomes, and minimal suitability expenses (Riseh and Vazvani, 2024). Hormone-dependent signaling pathways are recognized for their potential to influence defense mechanisms during mycorrhiza formation (Pozo and Azcón-Aguilar, 2007). This mechanism, referred to as mycorrhiza-induced resistance, has the potential to “prime” plants, enabling them to respond to an impending stressor, such as a pathogen attack, with enhanced speed and strength. Arbuscular mycorrhizal fungi (AMF) and *Trichoderma* spp. represent two beneficial microorganisms frequently utilized as plant biostimulants to enhance crop yields, as they promote plant growth and mitigate rhizospheric infections (Chaithra et al., 2025). Alongside promoting plant growth, AMF enhances photosynthesis and boosts resilience against pests, diseases, and abiotic stress (Hashem et al., 2025). *Trichoderma* species enhance plant health by producing enzymes that break down fungal cell walls, thereby improving crop nutrition, growth, and stress resilience while triggering resistance mechanisms (Philip et al., 2024). Our analysis of the findings concerning these standards suggests that tomato plants can develop tolerance to PVY infection through mycorrhization and the use of *Trichoderma* spp. applications. Under greenhouse conditions, the foliar application of *T. viride* isolate Tvd44 on tomato leaves and the colonization of tomato roots with AMF before PVY inoculation reduced the mitigation of viral effects. Remarkably, the combined application (dual+PVY) significantly reduced disease incidence, disease severity, and viral accumulation levels by 40%, 53.4%, and 88.1%, respectively. The findings align with recent studies indicating that the use of AMF effectively manages zucchini yellow mosaic virus (ZYMV) in cucumber plants, leading to a reduction in both disease incidence and severity compared to untreated plants (Metwally and Abdelhameed, 2024). In the same context, Khoshkhatti et al. (2020) reported that at 20 dpi, tomato plants treated with *Rhizoglomus irregularis* significantly mitigated the symptoms of tomato bushy stunt virus (TBSV) compared to the untreated ones. Furthermore, Miozzi et al. (2020) noted that the colonization of *Funneliformis mosseae* influenced the tomato’s susceptibility to cucumber mosaic virus (CMV) infection at 14 dpi. Moreover, Aseel et al. (2023b) showed that *T. viride* activates the host’s innate immune response and/or initiates systemic acquired resistance, potentially diminishing PVY and/or inhibiting its accumulation. Maffei et al. (2014) noted that tomato plants colonized by AMF and infected with the tomato yellow leaf curl Sardinia virus (TYLCSV) exhibited reduced symptoms and a lower viral titer. Nonetheless, the colonization by AMF was insufficient to mitigate the reduction in root biomass caused by the virus.

The current study employed microscopic examination to reveal the various mycorrhizal structures present in the roots of tomatoes across all treatments colonized by AMF. Miozzi et al. (2020) observed that AMF colonization in virus-inoculated plants resulted in a slight increase in mycorrhization density and the ratio of arbuscules throughout the entire root system compared to healthy plants. Our finding aligns with a recent study demonstrating that AMF effectively colonizes plant roots, as observed through a light microscope (Metwally and Abdelhameed, 2024). Additionally, Khoshkhatti et al. (2020) found that the mean colonization ratio of mycorrhiza-inoculated plants before TBSV/ToMV infection was not significantly different from that of non-infected control plants with AMF. The findings from our treatments for AMF+PVY, Tvd44+PVY, and dual+PVY treatments demonstrated a significant enhancement in plant growth evaluation when compared to tomatoes that were solely inoculated with PVY. Furthermore, another study showed that under greenhouse conditions, AMF-root colonization significantly boosted all growth measurements compared to other treatments, with increases observed in shoot length, dry weight, and fresh weight even when compared to healthy plants that weren’t colonized (Metwally and Abdelhameed, 2024; Gaši et al., 2025; Subhash et al., 2025).

Plant viruses can infiltrate the intracellular spaces of plant cells, where they closely associate with cellular organelles and the cytoplasm, leading to oxidative stress (Abdelkhalek et al., 2025). Plants possess defense mechanisms that incorporate antioxidant enzymes within their cells, thereby shielding them from oxidative stress (Rabie et al., 2024). These enzymes play a crucial role in regulating reactive oxygen species (ROS) and mitigating oxidative stress to lipids, proteins, and nucleic acids. Moreover, PVY infection resulted in a reduction in photosynthetic pigments (chlorophyll a and b) and antioxidant enzymes, such as CAT, while simultaneously increasing POX levels and decreasing total phenolic content. Furthermore, these parameters were notably influenced by increased enhancements resulting from AMF root colonization and TVD44 across all treatments, including AMF+PVY, TVD44+PVY, and the dual+PVY treatments. Similarly, it was noted that tomato plants treated with *T. viride* and *T. harzianum*, when infected with ToMV, exhibited increased levels of chlorophyll (a and b) and enhanced CAT activity compared to healthy tomato plants inoculated with ToMV (Aseel et al., 2024). In a similar context, Venkatesan et al. (2010) noted that the presence of chlorotic and necrotic symptoms in all virus-infected plants correlates with a reduction in the net photosynthetic rate and chlorophyll levels. The viral infection leads to damage and aggregation of chloroplasts, ultimately causing the destruction or halt of chloroplast synthesis (Abdelkhalek et al., 2019b). The characteristics of the host plant, the specific virus isolate, environmental conditions, and the progression of disease development all contribute to the extent of photosynthetic suppression (Akbar et al., 2021). AMF induces a physiological state that enhances plants’ ability to react more swiftly and effectively to pathogen assaults. Aseel et al. (2019) demonstrated that tomato plants colonized by AM fungi exhibit reduced susceptibility to the tomato mosaic virus.

Concerning the effect of the treatments tested on PVY titer, our findings indicated that the application of AMF+PVY, Tvd44+PVY, and dual+PVY treatments resulted in a notable decrease in *PVY-CP* expression in the leaves of tomato plants. The findings are consistent with previously reported data, indicating that AMF-colonized plants can reduce PVY symptom severity and lower viral accumulation levels (Deja-Sikora et al., 2024). Similar results showed that using *Trichoderma* significantly lowered the viral accumulation levels in the treated plant tissues (Thiem et al., 2014; Aseel et al., 2023b). The application of AMF and Tvd44 resulted in a significant upregulation of all eleven genes studied across various pathways, including WRKYs, JA, SA, flavonoid, and chlorogenic acid. *CHS* serves as the first enzyme in the flavonoid pathway, and the primary metabolites it produces are essential for flavonoid synthesis across different plant tissues (André et al., 2009). A previous investigation revealed a notable buildup of isoflavonoid and flavonoid compounds exhibiting diverse antibacterial properties against various phytopathogens linked to the overexpression of *CHS* (Dao et al., 2011; Martínez et al., 2017), as well as the plant’s response to viral infections and vice versa (Niggeweg et al., 2004; Abdelkhalek et al., 2018). Chlorogenic acid helps plants defend against diseases and prevent infections, including those caused by viruses (Abdelkhalek et al., 2022b; Salmerón et al., 2025). We suggest that the increased transcription levels of *CHS* signify their antiviral characteristics, illustrating that tomato plants can utilize polyphenolic compounds as a unique defense mechanism against viral infection and spread.

A gene expression network was used to illustrate the pathway containing these genes, in which *WRKY* transcription factors (such as *WRKY-1* and *WRKY-19*) control essential genes, including *CHS*, *C3H*, *F3’H*, and *FLS*, which in turn participate in the synthesis of flavonoids and other phenolic compounds crucial for defense. JERF factors also trigger defense responses through jasmonic acid signaling, which links to other plant immune responses (Roychowdhury et al., 2025). The detoxification of toxic chemicals and direct protection against infections are two functions of *GSTs* and *CHI* genes (Ellwanger et al., 2025). For instance, *WRKY-TFs* physically interact with proteins involved in plant defense, signaling, transcription, and other cellular processes to control and regulate plant signaling (Wani et al., 2021; Wang et al., 2025). The role of transcriptional factors from the WRKY family in plant virus infection remains inadequately explored. To fine-tune the defense response against various stresses, the defense signaling network, comprising numerous defense-associated genes, collaborates with *WRKY* members or other signaling proteins to regulate the overall expression of stress-responsive genes through autoregulation, cross-regulation, or protein-protein interactions (Freeborough et al., 2021). In this investigation, *CHS* gene is the master key in the physiological and pathway relationships of the genes (*F3’H*, *HQT*, *C3H*, *GST*, *JERF*, *CHI*, *WRKY-1*, *WRKY-19*, *FLS*, and *F3H*) in plant defense, which likely involves a complex network of interactions that regulate various aspects of plant defense responses, including the biosynthesis of flavonoids and other secondary metabolites and the activation of defense-related transcription factors. Overall, these genes create an interconnected system that governs the synthesis of secondary metabolites such as flavonoids and the management of defense response mechanisms, thereby enhancing the plant’s capacity to withstand pathogens and environmental stressors.

## 5 Conclusions

The current study investigated the impact of pre-inoculating tomato plants with arbuscular mycorrhizal fungi (AMF) and foliar application of *T. viride* against PVY. The colonization of AMF and the foliar application of *T. viride* significantly enhanced the tolerance of tomato plants to PVY by inducing systemic resistance and increasing the expression of defense-related genes and enzymes. The results indicated that the treatments improving the efficiency of defense responses in plants during pathogen attacks engage pathways governed by jasmonic acid (JA), salicylic acid (SA), and flavonoids. Consequently, treatments with AMF and *T. viride* alleviated the viral effects on the physiological and transcriptional disturbances induced by PVY infection. The symbiotic relationship between mycorrhizal fungi and most land plants suggests that these fungi, along with *T. viride*, could be viable, eco-friendly options for controlling viral diseases in crops.

## Data Availability

The original contributions presented in the study are included in the article/**Supplementary Material**. Further inquiries can be directed to the corresponding authors.
