# Supplementary material for: Impacts of arbuscular mycorrhizal and Trichoderma viride on enhancing physicochemical properties and triggering defense mechanisms of tomato plants challenged with potato virus Y
Source: Front Plant Sci. 2025 Aug 22;16:1650871. doi: 10.3389/fpls.2025.1650871 (PMC12411435; doi:10.3389/fpls.2025.1650871)
Supplement: Supplementary file 1 [file Table1.docx]

**Table S1**. Primer sequences of defense pathway genes used in RT-qPCR assay.

| **Gene name** | **Abbreviation** | **Primer Sequence (5**′**–3**′**)** | **Pathway** |
| --- | --- | --- | --- |
| WRKY transcription factor 1 | *WRKY1-F* | CGTGCAGCAGCAAAGCAA | SbWRKY transcription factors |
|  | *WRKY1-R* | GTCGCAGGTATGCTCGTTGA |  |
| WRKY transcription factor 19 | *WRKY19-F* | AATGTCCCTCTGGCGAACTC |  |
|  | *WRKY19-R* | CAGTACACCCAAGGCTCCAT |  |
| Jasmonate and ethylene-response factor 3 | *JERF3-F* | GCCATTTGCCTTCTCTGCTTC | JA/ET-signaling pathways |
|  | *JERF3-R* | GCAGCAGCATCCTTGTCTGA |  |
| Glutathione S-transferase 1 | *GST1-F* | CGGTGACTTGTACCTCTTCGAATC | SA-binding receptor proteins |
|  | *GST1-R* | ATCCACCATTGCTGCCTCC |  |
| Flavanone 3-hydroxylase | *F3H-F* | CCAAGGCATGTGTGGATATGG ACC | Flavonoid biosynthetic pathway |
|  | *F3H-R* | CCTGGATCAGTATGTCGTTCAGCC |  |
| Chalcone synthase | *CHS-F* | CACCGTGGAGGAGTATCGTAAGGC |  |
|  | *CHS-R* | TGATCAACACAGTTGGAAGGCG |  |
| Flavonol synthase 1 | *FLS1-F* | CCTCCTTCCTACAGGGAAGCAAA |  |
|  | *FLS1-R* | CAAGCCCAAGTGACAAGCTCCTAA |  |
| Chalcone isomerase 2 | *CHI2-F* | GGCAGGCCATTGAAAAGTTCC |  |
|  | *CHI2-R* | CTAATCGTCAATGATCCAAGCGG |  |
| Flavonoid 3′ hydroxylase | *F3´H-F* | TGGGTATACCCAAACTCATTCCG |  |
|  | *F3´H-R* | AAAAGCCCAAAGTTGATGTGAAAG G |  |
| Hydroxycinnamoyl-CoA quinate transferase | *HQT1-F* | CCCAATGGCTGGAAGATTAGCTA | Chlorogenic biosynthetic pathway |
|  | *HQT1-R* | CATGAATCACTTTCAGCCTCAACAA |  |
| p-coumarate 3-hydroxylase | *C3H-F* | TTG GTG GCTACGACATTCCTAAGG |  |
|  | *C3H-R* | GGTCTGAACTCCAATGGGTTATTC C |  |
| Potato virus Y | *PVY-CP-F* | CAACTCCAGATGGAACAATTG | Coat protein |
|  | *PVY-CP-R* | CCATTCATCACAGTTGGC |  |
| *Beta-actin* | *β-actin-F* | ATGCCATTCTCCGTCTTGACTTG | Housekeeping |
|  | *β-actin-F* | GAGTTGTATGTAGTCTCGTGGATT |  |
